# Elimination characteristics of post-operative isoflurane levels in alveolar exhaled breath via PTR-MS analysis

**DOI:** 10.1088/1752-7155/10/4/046006

**Published:** 2016-10-12

**Authors:** R Fernández del Río, M E O’Hara, P Pemberton, T Whitehouse, C A Mayhew

**Affiliations:** 1 Molecular Physics Group, School of Physics and Astronomy, University of Birmingham, Birmingham B15 2TT, UK; 2 Critical Care and Anaesthesia, University Hospital Birmingham NHS Trust, Birmingham, B15 2TH, UK; 3 Breath Research Institute, Leopold-Franzens University of Innsbruck, 6850 Dornbirn, Austria; r.fernandezdelrio@bham.ac.uk

**Keywords:** proton transfer reaction mass spectrometry, isoflurane anaesthesia, alveolar breath analysis, cognitive function, volatile organic compounds

## Abstract

Isoflurane (1-chloro-2,2,2-trifluoroethyl difluoromethyl ether), C_3_H_2_ClF_5_O, is a commonly used inhalation anaesthetic. Using a proton transfer reaction mass spectrometer (PTR-MS) we have detected isoflurane in the breath of patients several weeks following major surgery. That isoflurane is detected in the breath of patients so long after being anaesthetised raises questions about when cognitive function has fully returned to a patient. Temporal profiles of isoflurane concentrations in breath are presented for five patients (F/M 3/2, mean age 50 years, min–max 36–58 years) who had undergone liver transplant surgery. In addition, results from a headspace analysis of isoflurane are presented so that the product ions resulting from the reactions of H_3_O^+^ with isoflurane in PTR-MS could be easily identified in the absence of the complex chemical environment of breath. Six product ions were identified. In order of increasing *m*/*z* (using the ^35^Cl isotope where appropriate) these are $\text{CHF}_{2}^{+}$ (*m*/*z* 51), CHFCl^+^ (*m*/*z* 67), CF_3_CHCl^+^ (*m*/*z* 117), C_3_F_4_OCl^+^ (*m*/*z* 163), C_3_H_2_F_4_OCl^+^ (*m*/*z* 165), and C_3_F_4_OCl^+^ H_2_O (*m*/*z* 183). No protonated parent was detected. For the headspace study both clean air and CO_2_ enriched clean air (4% CO_2_) were used as buffer gases in the drift tube of the PTR-MS. The CO_2_ enriched air was used to determine if exhaled breath would affect the product ion branching ratios. Importantly no significant differences were observed, and therefore for isoflurane the product ion distributions determined in a normal air mixture can be used for breath analysis. Given that PTR-MS can be operated under different reduced electric fields (*E*/*N*), the dependence of the product ion branching percentages for isoflurane on *E*/*N* (96–138 Td) are reported.

## Introduction

In a recent paper we reported a study of the volatile biomarkers in breath associated with liver disease using proton transfer reaction mass spectrometry (PTR-MS) [1]. The results of that study strongly suggest that three volatiles, methanol, 2-pentanone and limonene, are related to a diseased liver. The risk of false discovery was minimised by using a two stage process to reduce the variable set so that we did not have to rely on unsupervised multivariate analysis. Importantly, for this present work 12 of the 31 patients investigated in the first stage, which involved comparing breath samples of patients suffering with chronic liver disease with 30 healthy controls (usually partners of the ill patients), had their breath analysed post liver transplant. Of these 12 patients, 5 were followed longitudinally as in-patients during the post-transplant period for a number of weeks. For all 12 patients, a number of ions were observed in the proton transfer reaction mass spectra of the post-transplant breath samples, which were not present in the pre-transplant breath samples. We considered that these result from a reaction of H_3_O^+^ with the anaesthetic, isoflurane, (C_3_H_2_ClF_5_O) used during surgery. In agreement with this, one of the ions that we observe at *m*/*z* 165, has been assigned to be $\text{C}{{\text{F}}_{\text{2}}}\text{HOCHClCF}_{2}^{+}$ from a Selected Ion Flow Tube-MS (SIFT-MS) analysis of the headspace of isoflurane with H_3_O^+^ as the reagent ion [2].

Given our longitudinal measurements, which provide mass spectra of post-transplant breath samples for several weeks after the operation, we had a unique opportunity to investigate how long isoflurane remains in the body of unhealthy patients following major surgery. This may have consequences in terms of the intellectual function of patients by providing details on how quickly patients can safely return to a normal life.

Other PTR-MS studies have either concentrated on monitoring an intravenous anaesthetic, propofol, during surgery [3–8] or for monitoring sevoflurane and isoflurane in the hospital environment, namely in an urological post-anaesthesia care unit [9] and for sevoflurane in an operating theatre [10].

There have been several other studies investigating the elimination of volatile anaesthetics from the body following surgery. Yasuda *et al* [11] used gas chromatography with flame ionisation detection (GC-FID) to compare the kinetics of sevoflurane and isoflurane in humans. Of relevance to our study they report a fast elimination of isoflurane from the body, namely within a day the breath concentrations had decreased by an order of magnitude. However, their work used seven healthy male volunteers (mean age of 23 years with a standard deviation of 3 years). Therefore, the rapid decrease in concentration may not be a true reflection of what happens with sick, obese and/or older people. A more recent paper by Ghimenti *et al* [12] reports details on a study using GC-MS of post-operative elimination of sevoflurane and its metabolite, hexafluroisopropanol, in exhaled breath of six very sick patients that had undergone a variety of surgeries. They used pharmokinetic models for assessing liver function. In their investigations mixed-expired breath samples (multiple deep breaths) were collected in disposable Nalophan bags. The samples were then transferred to desorption tubes for subsequent analysis. Here we report the first PTR-MS longitudinal study investigating isoflurane in the breath of patients following surgery. Prior to being able to analyse the mass spectra of the post-transplant breath samples, it was necessary to establish which product ions result from the reaction of H_3_O^+^ with isoflurane in the drift tube environment of a PTR-MS, divorced from the complex chemical environment of breath. Therefore, as part of this study we present a headspace analysis of isoflurane at the reduced electric field of 136 Td used in our previous liver study. Given that the product ions found in this study differ from those by Wang *et al* [2], we also present the product ion branching ratios over an extended reduced electric field from approximately 90 Td through to 140 Td. These have been determined using both clean air and CO_2_ enriched clean air, to mimic breath, as the buffer gases in the drift tube of our PTR-MS. The use of CO_2_ enriched air is important because the presence of a high percentage of CO_2_ could influence the proton transfer reaction leading to different product ion distributions, which need to be taken into account when analysing breath samples [13, 14].

## Experimental details and methods

### Headspace analysis

Isoflurane (CAS Number 26675-46-7) was purchased from Sigma Aldrich (UK).

Isoflurane samples were prepared for analysis by adding 1 *µ*l of isoflurane into a 100 ml glass bottle. A 100 ml glass syringe was then coupled to the top part of the bottle using a 3-way luer-lock stopcock and 10 ml of the isoflurane headspace gas was sampled. The isoflurane sample was then diluted in the glass syringe with 90 ml of clean air or CO_2_ enriched clean air (4% carbon dioxide, 17% oxygen and 79% nitrogen (Scientific and Technical Gases Ltd (STG, Staffordshire, UK)). The concentration of the isoflurane was sufficiently low that no depletion of the H_3_O^+^ signal was observed.

### Patients

Patients were recruited at the University Hospital Birmingham from the transplant assessment clinic. Of the 31 patients who consented to take part in the clinical trial, 12 went on to have liver transplant. Of these we were able to follow five patients for several weeks following surgery (F/M 3/2, mean age 50 years, min–max 36–58 years). Table 1 summarises the details of these five patients, with each patient being just identified by sex (F or M) and a number, which was used in our previous study and retained here for comparison [1]. Note that the number of samples and days at which breath samples were taken varies because of the availability and health of the patient. Patients were followed from the Intensive Care Unit after their transplant, through to the wards until their discharge. Some patients recovered and were discharged quicker than others so there are fewer data points for them.

**Table 1.** Liver transplant patient details, including sex (female F, male M), age, location of post-transplant breath sampling (Out-Patient Clinic (OPC) or Ward), and number of days after transplant when breath samples were collected.

Patient ID	Age (year)	Location of post-transplant breath sample	Post-transplant breath samples: days after transplant
F2	49	OPC	3, 5, 130
F4	58	Ward	5–8, 11–15, 18, 58
F5	53	Ward	2–6, 9–12
M3	53	Ward	4, 7, 48
M7	36	Ward	2, 3, 6–8, 55

In addition to taking breath samples from controls as described in [1], samples of air in the locations where the patients were in, were collected so that allowances for any background isoflurane could be taken into account.

Hospital room air was collected every time breath samples were taken using glass syringes (2 syringes for every sample), which were analysed in the same way as the breath samples. Our data relating to isoflurane concentrations in the hospital environment show that the maximum concentration for any of the sampling days was at 4 ppbv. There was one exception to this when 13 ppbv was measured for one patient, F4, who was in a small enclosed room with poor ventilation, and therefore the patient was almost certainly contaminating the air with her own breath. Thus any contribution to the isoflurane signal from the environment is negligible compared to that in the breath.

### Breath sampling protocol

A detailed description of the breath sampling protocol used can be found in our earlier paper dealing with volatiles associated with liver disease [1]. In brief, capnography controlled sampling was used to collect only the alveolar phase of the breath. Subjects were seated and in a relaxed state and were asked to breathe normally into a gas tight respiratory system (Intersurgical Limited) containing an in-line CO_2_ mainstream sensor connected to a fast-time response capnometer (Capnogard 1265 Novametrix Medical Systems Inc.). A 100 ml glass syringe (Sigma-Aldrich) was coupled to the tubing using a 3-way luer-lock stopcock (Braun Medical Limited). When the alveolar plateau on the capnograph was observed, the breath sample was manually drawn from the subject’s breath stream into the syringe. Three to four breaths samples were collected into each 100 ml syringe, and four replicates of these were taken for each subject. Glass syringes were used, because our tests showed that they minimize surface adsorption. We avoided the use of bags owing to potential problems associated with losses and ageing effects [15].

Within two hours after collection, breath samples were mass spectrometrically analyzed by PTR-MS, for which the syringes were maintained at a constant temperature of 40 °C using a heating bag (Infroheat, Wolverhampton). This was done to limit condensation, which could otherwise lead to volatile loss [16]. The outlet from each syringe was connected directly to the inlet of the PTR-MS. Within two hours we have found that there is no significant loss of isoflurane in the syringe.

### Analytical measurements

PTR-MS is a technology designed to detect low concentrations of volatiles (less than parts per billion by volume). Hence it has found use in many analytical applications ranging from drug detection through to industrial pollution [17]. Details of the instrument used, a first generation PTR-Quad-MS (IONICON Analytik GmbH), and how it operates are described in detail in the literature [17, 18]. In brief, it exploits the reactions of protonated water with neutral volatiles (M), often leading to a protonated parent (MH^+^). If dissociative proton transfer occurs then it is not extensive in terms of the number of resulting product ions. The drift tube was maintained at 2.07  ±  0.01 mbar and temperature of 45  ±  1 °C. For the breath analysis the drift-tube voltage was set at the standard recommended value of 600 V. For the headspace analysis the voltage across the drift-tube was changed from 400 V to 600 V at intervals of 20 V, *E*/*N* (96–138 Td).

Mass spectra of the breath samples were recorded and the areas under the spectral peaks converted to a volume mixing ratio (VMR) by use of a standard procedure that relies on the use of an experimentally measured reaction rate coefficient and reagent and product ion counts [17].

## Results and discussion

### Headspace analysis of isoflurane

No protonated isoflurane (*m*/*z* 185) was observed for any value of the reduced electric field. This agrees with the results of Wang *et al* who conclude that once formed the excited protonated parent spontaneously dissociates via various pathways [2]. However, in comparison to Wang *et al* SIFT-MS (thermal energy) measurements, we find substantial differences in product ions and their branching ratios. In our measurements we have observed primary product ions at *m*/*z* 51, *m*/*z* 67, *m*/*z* 117, *m*/*z* 163, and *m*/*z* 165. By taking into account isotopic abundance we have assigned these to be $\text{CHF}_{2}^{+}$, CHFCl^+^, CF_3_CHCl^+^, C_3_F_4_OCl^+^, and C_3_H_2_F_4_OCl^+^, respectively. Another ion involving isoflurane is observed at *m*/*z* 181 which is assigned as a secondary (association) reaction of C_3_F_4_OCl^+^ with H_2_O. The product ion percentage branching ratios at a commonly used reduced electric field of 138 Td are presented in table 2 and compared to the measurements of Wang *et al*.

**Table 2.** *m*/*z* values (dominant ion peak), proposed product ions and branching ratio percentages resulting from the reaction of H_3_O^+^ with isoflurane using PTR-MS (reduced electric field of 138 Td) and SIFT-MS [2].

*m*/*z*	Proposed production	% PTR-MS	% SIFT-MS [2]
51	$\text{CHF}_{2}^{+}$	12	—
67	CF_2_HO^+^	—	15
67	CHFCl^+^	50	—
99	CF_3_CH_2_O^+^	—	10
117	CF_3_CHCl^+^	6	30
119	$\text{C}{{\text{F}}_{\text{3}}}\text{CFHOH}_{2}^{+}$	—	25
147^a^	CF_3_HOCHClCH^+^	—	10
163	CF_3_CClOCF^+^	24	—
165	$\text{C}{{\text{F}}_{\text{2}}}\text{HOCHClCF}_{2}^{+}$	8	10

a In the paper by Wang *et al* the ion at *m*/*z* 147 is incorrectly given as CF_2_HOCHClCH^+^.

*Note*: The percentages have taken into account the ^37^Cl isotope for the chlorine containing ions. For the PTR-MS studies the product ion branching percentages have an error of approximately  ±20%.

Wang *et al* observed no *m*/*z* 51 ions. This almost certainly results from the differences in collisional energies and possibly the internal energy of the H_3_O^+^ ions, both of which are considered to be at thermal energies in SIFT-MS systems. Certainly we only observe a significant branching ratio for this ion at 138 Td. The supplementary information (stacks.iop.org/JBR/10/046006/mmedia) provides details on the product ion branching ratios as a function of reduced electric field. This is provided because different PTR-MS users use different reduced electric fields and also because of the major differences observed in the product ions and branching ratios compared to the only other headspace analysis by Wang *et al* also observed product ions at *m*/*z* 67, but assign it to be CF_2_HO^+^ rather than CHFCl^+^, *m*/*z* 99 (assigned to be CF_3_CH_2_O^+^), which we did not observe, *m*/*z* 117 (CF_3_CHCl^+^, in agreement with our assignment), *m*/*z* 119 ($\text{C}{{\text{F}}_{\text{3}}}\text{CFHOH}_{2}^{+}$), which we do not observe other than the ^37^Cl isotope of CF_3_CHCl^+^, *m*/*z* 147 (CF_2_HOCHClCH^+^), which we do not observe, and *m*/*z* 165 (C_3_H_2_F_4_OCl^+^).

Given that the product ion branching ratios were obtained using normal air in the drift tube, a question remains as to whether the presence of higher CO_2_ concentrations in breath could affect these ratios through thermalisation effects as a result of collisions of H_3_O^+^ with CO_2_ prior to reaction with isoflurane. This is an important issue for breath analysis using PTR-MS. Keck *et al* [14] have already demonstrated that higher CO_2_ concentrations in the drift tube buffer gas of a PTR-MS enhances the concentration ratio of the protonated water dimer to protonated water and changes the mass spectra of key breath gases such as methanol, ethanol, 1-propanol, 2-propanol, acetone and isoprene. We therefore undertook a headspace analysis using CO_2_ enriched (4%) air in the drift tube to determine if the product ion distributions associated with isoflurane would be altered when analysing the breath samples. We find that within experimental uncertainty no significance difference in product ion percentage branching ratios is observed between normal and CO_2_ enriched air buffer gases (see supplementary information). There is still a question of changes in humidity [2], but that will be the subject of another study.

### Longitudinal measurements of isoflurane in breath samples post liver transplant

By using the product ions and their branching ratios determined in the headspace analysis we can determine the isoflurane concentrations in breath by adding the intensities of all product ions that result from the reaction of H_3_O^+^ with isoflurane at a reduced electric field of 138 Td, namely *m*/*z* 51, *m*/*z* 67 (69), *m*/*z* 117 (119), *m*/*z* 163 (165), and *m*/*z* 165 (167). It should be noted that the *m*/*z* 69 signal intensity cannot be used directly owing to the presence of isoprene in human breath. Therefore the total ion signal associated with CHFCl^+^ is determined only from the *m*/*z* 67 ion intensity. However, it is important to point out that since isoflurane in breath results in a spectral peak at *m*/*z* 69 (CHF^37^Cl^+^) there are consequences for any PTR-MS studies which monitor isoprene in the breath following a surgical procedure during which isoflurane is used. The contribution of isoflurane to the signal intensity at *m*/*z* 69 was found to be significant and varied between 12% to 99% at the beginning of our longitudinal study, being dependant on the concentration found in a patient’s breath.

**Figure 1.**
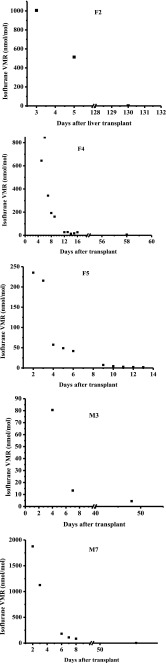
Longitudinal changes in volume mixing ratios (VMR) in nmol/mol for isoflurane for given days after liver transplant for patients F2, F4, F5, M3, and M7. Error bars are not shown as they are smaller than the data points.

Figure 1 shows the temporal changes in isoflurane concentrations for the five patients: F2, F4, F5, M3 and M7. The important observation is that we did not observe rapid decreases in isoflurane concentrations following surgery. This is different from the results of Yasuda *et al* who found that the elimination of isoflurane from the human body was fast [10], i.e. within one day the signal intensity had been observed to have dropped by about an order of magnitude, whereas in our study we still found high concentrations of isoflurane in the breath several days after the operation. Although the Yasuda *et al* study used healthy young male subjects (age 23  ±  3 years (mean  ±  sd)), the observed temporal difference is still surprising given that the major elimination of isoflurane is through breath. Isoflurane is poorly metabolised (0.17%) by hepatic cytochrome P450 enzymes in the liver [19].

A possible explanation for the observed difference between our results and those of Yasuda *et al* could be associated with the physical condition of the subjects/patients involved and the duration of isoflurane administration. Yasuda *et al* volunteers were healthy and had a mean body mass index (BMI) of 21.7 kg m^−2^, i.e. all were within the normal (healthy weight) range. In comparision F2, F4, F5, M3 and M7 had BMIs of 31.2, 32.4, 28.0, 32.5, and 35.0 kg m^−2^, respectively. Thus our patients were either overweight or obese. Furthermore, Yasuda *et al* administered isoflurane for precisely 30 min, whereas our patients underwent surgeries that lasted between 5 to 7 h. Although isoflurane has a low blood/gas partition coefficient of 1.45  ±  0.12 (mean  ±  SD) [20], it has a high oil/blood partition coefficient of 97, and therefore we propose that for our patients a significant amount of the isoflurane was absorbed into fat tissue, only to be slowly released after surgery. In support of this argument, it has been found that if isoflurane is administered for more than 2 h, as would be the case for our patients, the adipose tissue becomes saturated and then the emergence from the anaesthetic is prolonged [21].

Finally, we comment that of all our patients, patient M7 showed the fastest elimination of isoflurane even though he had the highest concentration in exhaled breath after surgery and was the most obese patient. However, the elimination was still much less than found in the study of Yasuda’s study. The rapid washout, in comparison to the other patients, could be a result of this patient being the youngest of the group involved at 36 years old. (F2, F4, F5 and M3 ages were 49, 58, 53 and 53 years, respectively.) M7 also recovered from surgery more quickly than the other patients in this group and was discharged within one week. However, with such a small number of patients we cannot state whether or not there is any correlation with age and perhaps gender with regards to isoflurane elimination from fatty tissue. More detailed studies are therefore required.

## Conclusions

An aim of this study was to determine which product ions result from the reaction of H_3_O^+^ with isoflurane in order to monitor isoflurane in the breath. No protonated parent was observed, instead the protonated parent rapidly fragments to diagnostically useful ions with *m*/*z* values of 67 (69), 117 (119), 163 (165) and 165 (167), which are assigned to be CHFCl^+^, CF_3_CHCl^+^, C_3_F_4_OCl^+^ and C_3_H_2_F_4_OCl^+^ respectively. Using these ions, the wash out characteristics of isoflurane following liver transplant have shown that a patient will still have high levels of isoflurane in their blood even several days after the operation. This could have an impact on a patient’s ability to drive and use machines after isoflurane anesthesia administration, especially after a long operation, when isoflurane will be stored in fat tissue.
